# Epidemiological, Clinical and Analytical Features in Lyme Borreliosis Patients Seropositive for *Babesia divergens/venatorum*

**DOI:** 10.3390/microorganisms13061383

**Published:** 2025-06-13

**Authors:** María Folgueras, Luis Miguel González, Aitor Gil, Julio Collazos, Mercedes Rodríguez-Pérez, Laura Pérez-Is, Javier Díaz-Arias, María Meana, Belén Revuelta, Jeremy Gray, Estrella Montero, Víctor Asensi

**Affiliations:** 1 Infectious Diseases Unit, Hospital Universitario Central de Asturias, Oviedo University School of Medicine, Avda Roma s/n, 33011 Oviedo, Asturias, Spain; mariafolgueras@gmail.com (M.F.); vasensia@gmail.com (V.A.); 2 Microbiology and Infectious Diseases Group, Instituto de Investigación Sanitaria del Principado de Asturias (ISPA) Oviedo, Avda Roma s/n, 33011 Oviedo, Asturias, Spain; 3 Parasitology Reference and Research Laboratory, Centro Nacional de Microbiología, Instituto de Salud Carlos III, Majadahonda, 28220 Madrid, Spain; lmgonzal@isciii.es (L.M.G.); aitor.gil@edu.uah.es (A.G.);; 4 Infectious Diseases Unit, Hospital de Galdácano, Labeaga Auzoa, 48960 Galdácano, Vizcaya, Spain; 5 Microbiology Service, Hospital Universitario Central de Asturias, Oviedo University School of Medicine, Avda Roma s/n, 33011 Oviedo, Asturias, Spain; 6 Internal Medicine Service, Hospital Vital Álvarez-Buylla, Vistalegre, 2, 33611 Mieres, Asturias, Spain; 7 UCD School of Biology and Environmental Science, University College Dublin, D04 N2E5 Dublin, Ireland; jeremy.gray@ucd.ie

**Keywords:** *Babesia divergens*/*venatorum*, babesiosis, *Borrelia burgdorferi*, cardiorespiratory symptoms, Lyme borreliosis, myocardial damage

## Abstract

Lyme borreliosis (LB), caused by *Borrelia burgdorferi* sensu lato (s.l.) and babesiosis, caused by *Babesia divergens* and *Babesia venatorum*, are both transmitted by the ixodid tick *Ixodes ricinus*. Although these diseases coexist in Spain and other European regions, no studies have been carried out to assess the impact on the health of patients exposed to both causative pathogens. This retrospective study, conducted in Asturias in northwestern Spain between 2015 and 2017, analyzed the possible complications arising from exposure to these pathogens. To this end, the epidemiological, clinical and analytical features of two groups of a cohort of 120 LB patients exposed to one or two of the pathogens were compared. The cohort comprised 73 patients who had only been infected with *Bo. burgdorferi* s.l. (Bb group) and 47 patients who were seropositive for *Ba. divergens*/*venatorum* in addition to being infected with *Bo. burgdorferi* s.l. (BbBdv group). The results showed that LB patients who had also been exposed to infection with *Babesia* spp. (BbBdv group) had significantly more cardiorespiratory symptoms, in particular dyspnea and first- and second-degree atrioventricular (AV) blocks, compared to those who had been infected with *Bo. burgdorferi* s.l. alone (Bb group). No relevant differences in other symptoms, epidemiological factors or analytical tests were observed between the two groups.

## 1. Introduction

In recent years, tick-borne diseases (TBDs) have increased significantly worldwide [1]. One of the most common TBDs affecting humans is Lyme disease, also known as Lyme borreliosis (LB) [2]. Another TBD, babesiosis, is relatively rare but is frequently life-threatening, especially in immunocompromised patients [3,4]. The commonest and most widespread tick in Europe, *Ixodes ricinus*, transmits the causative pathogens of both diseases. The spirochaete *Borrelia burgdorferi* sensu lato (s.l.) complex is responsible for LB and utilizes a wide range of small mammals and birds as reservoir hosts. Typical symptoms include erythema migrans, accompanied by fever, headache and fatigue. Without antibiotic treatment, the infection can spread to the joints, the heart and the nervous system [2]. European babesiosis is caused by the protozoan parasites *Babesia divergens*, *Babesia microti* and *Babesia venatorum*, which infect erythrocytes, giving rise to manifestations that include fever, chills, sweating, myalgias, fatigue, hepatosplenomegaly, and anaemia. The natural hosts of *Ba. divergens* are cattle, whereas *Ba. venatorum* is found naturally in roe deer (*Capreolus capreolus*) and causes a similar disease, but is considered to be less pathogenic and may cause low-level chronic infections [3,5]. It is detected more frequently than *Ba. divergens* in the vector tick, *I. ricinus* [6,7], especially in woodland habitats, which are commonly associated with LB [8]. A recent study in Northern Spain suggests that *Ba. venatorum* occurs quite frequently in *I. ricinus* [9] and also in roe deer [10]. Genotypes of *Ba. microti*, responsible for chronic and subclinical disease in the USA, have been detected in *I. ricinus* ticks in Europe, but there are evidently very few records of the pathogen in Spanish ticks and it rarely causes disease in Europe [3].

Serological studies indicate that a significant number of individuals in Europe have been exposed to *Babesia* parasites, with seroprevalences ranging from 2% to 39.7% [5,11,12,13,14]. The majority of the seroprevalence studies have been carried out using the indirect immunofluorescence assay (IFA), which can distinguish *Babesia microti* from *Ba. divergens* and *Ba. venatorum*, but since the latter two are antigenically similar, cross-reactivity between these two species occurs in IFA tests detecting IgG antibodies against *Ba. divergens* [3,14,15,16]. In the absence of molecular data, therefore, it is not possible to definitively attribute such infections to either *Ba. divergens* or *Ba. venatorum* [16].

Despite high seroprevalence rates, clinical cases of babesiosis are uncommon, so most *Babesia* infections in European populations are evidently asymptomatic or paucisymptomatic [3,4]. This is a relevant but underexplored point, especially in the context of subclinical and chronic infections caused by *Babesia* species other than *Ba. microti* such as *Ba. divergens* and *Ba. venatorum* [3,17]. In particular, it would be a significant advance to determine whether individuals who are usually considered asymptomatic for babesiosis could in fact harbor subclinical infections of *Ba. divergens* or *Ba. venatorum* that affect their health.

Simultaneous and consecutive LB/babesiosis infections are suspected in Europe, as both TBDs are transmitted by the same tick species [18,19]. The case of a patient in Finland, who developed a fatal infection with *Ba. divergens* co-infected with *Bo. burgdorferi* s.l., together with the serological evidence, suggests that Europeans are exposed to or infected with both *Bo. burgdorferi* s.l. and *Ba. divergens/venatorum* consecutively or simultaneously [12,14,20]. However, to date, no studies in Europe have analyzed the clinical progression of such multiple infections.

Coinfection with *Bo. burgdorferi* and *Ba. microti* is relatively common in some North American states, where the number of symptoms and duration of illness in patients with concurrent Lyme disease and babesiosis are higher than in patients with either infection alone [19,21,22,23,24]. However, differences in ixodid tick vectors, *Borrelia* and *Babesia* species and in prevalences and clinical presentations in the USA and Europe make direct comparisons difficult and encourage further research.

To shed more light on this question, we recently conducted a retrospective study in Asturias (northwestern Spain), which resulted from the recent occurrence of two cases of *Ba. divergens* babesiosis in the region [12]. This study showed that in a cohort of 120 patients infected with *Bo. burgdorferi*, 47 (39.2%) were also positive for *Ba. divergens/venatorum*, according to the IFA (BbBdv group). The remaining 73 patients were positive for *Bo. burgdorferi* s.l. alone (Bb group) [12]. In the present study, epidemiological, clinical, laboratory, imaging, electrocardiographic (ECG) and therapeutic characteristics observed in the Bb and BbBdv groups were compared to identify significant differences between LB patients exposed to *Ba. divergens*/*venatorum* and those without *Babesia* antibodies.

## 2. Materials and Methods

### 2.1. Description of the Patients and Study Designed

This study included 120 patients ≥18 years old infected with *Bo. burgdorferi* s.l. who resided, from 2015 to 2017, in 74 out of 78 councils of Asturias (northwestern Spain). In our previous study [12], patients were attending the Infectious Diseases, Neurology, Rheumatology and/or Dermatology Services of the Hospital Universitario Central de Asturias (HUCA), Oviedo, Spain, or other regional affiliated hospitals. All patients were clinically diagnosed with LB and initially categorized according to current guidelines for the period 2015–2017 [25], and then retrospectively adjusted using updated criteria [2,26]. Early localized LB (Stage 1) included patients with erythema migrans and a positive *Bo. burgdorferi* s.l. IgG and/or IgM serology within 1–28 days following a tick bite. Early disseminated LB (stage 2) included patients who developed multiple erythema migrans, neuroborreliosis or carditis within 3–12 weeks after the initial infection. Late persistent LB (stage 3) included patients with arthritis and acrodermatitis chronica atrophicans months to years after the initial infection. Patients with confirmed Lyme arthritis had asymmetrical, monoarticular or oligoarticular arthritis, synovial fluid with 10,000–25,000 cells/mm^3^ and synovial fluid-positive anti-*Borrelia* IgG antibodies, and positive *Borrelia* IgG and/or IgM serology. Patients with Lyme carditis typically experienced syncope, chest pain or dyspnea and had positive *Borrelia* IgG and/or IgM serology, with no other cause for their cardiac symptoms. Patients with neurological LB had compatible clinical symptoms and/or signs (limb paresthesia/paresis, gait disturbance, cranial neuritis and headache), cerebrospinal fluid (CSF) pleocytosis, and CSF-positive *Borrelia* IgG serology. Late-stage LB included patients with typical symptoms such as oligoarthritis primarily affecting large joints, especially the knees within months or years after the initial infection.

In our previous study [12], patients clinically diagnosed with LB were also serologically confirmed using commercial tests, Vidas (BioMerieux, Madrid, Spain) and Borrelia IgG IgM EcoLine (Sekisui Diagnostics, Rüsselsheim, Germany) [12,27] at the Microbiology Service of the HUCA. Moreover, IgG antibodies against *Ba. divergens/venatorum* were detected in the serum samples of 47 patients by IFA at the Centro Nacional de Microbiología, Instituto de Salud Carlos III, Madrid, Spain. Western blot using a *Ba. divergens* (Rouen 87 strain) protein extract as target, confirmed 85.1% of the positive serum samples detected by IFA. Moreover, 32% of the serum samples presented specific antibodies against the major surface protein of *Ba. divergens* (Bd37) by Western blot using Bd37 recombinant protein as a target [12]. The cut-off titres used for discrimination between seronegative and seropositive reactions by IFA and Western blot assays were set at 1:128 for *Ba. divergens*, following World Health Organization (WHO) guidelines [11,14].

In the current study, patients were classified into two different groups: those (*n* = 73) infected only with *Bo. burgdorferi* s.l. (group Bb) and those (*n* = 47) infected with *Bo. burgdorferi* s.l and seropositive for *Ba. divergens/venatorum* (group BbBdv). All patients’ electronic medical records were searched and their demographic, epidemiological, predisposing factors, clinical, laboratory, imaging, ECG and other data were collected and compared between the two groups.

### 2.2. Statistical Analysis

The continuous variables did not follow a Gaussian distribution, according to the Kolmogorov–Smirnov test and, therefore, non-parametric tests were used for analysis. The values were reported as medians (IQ range) or as percentages for continuous or categorical variables, respectively. Both, Bd and BbBdv groups were compared with the Mann–Whitney U test for continuous variables and the Chi-square test and the Fisher’s exact text, when appropriate, for categorical variables. A stepwise logistic regression analysis model was constructed to identify the factors independently associated with the diagnosis of double seropositivity. A *p* value < 0.05 for a two-tailed test was considered statistically significant. Calculations were carried out with the statistical software SPSS v. 25 (IBM Corp., Armonk, New York, NY, USA).

## 3. Results

This study included 120 patients diagnosed with LB. According to Montero et al. [12], 73 of these patients, were infected with *Bo. burgdorferi* s.l. only (group Bb), while 47 were infected with *Bo. burgdorferi* s.l. and were seropositive for *Ba. divergens/venatorum* (BbBdv group). Table 1 shows that positive *Bo. burgdorferi* s.l. IgG serology was observed in all patients by definition and *Bo. burgdorferi* IgM serology was positive in 58.3%. *Babesia divergens/venatorum* IgG antibodies were detected by IFA in 39.2% (47/120) of the serum samples from patients infected with *Bo. burgdorferi* s.l. In addition, Western blot using *Ba. divergens* protein extracts confirmed 85.1% of the positive serum samples detected by IFA and 32% of the serum samples presented specific antibodies against the *Ba. divergens* major surface antigen, Bd37 [12,28].

The median age of the patients was 57.2 years (IQ range 45.5–71.8) and 81 patients (67.5%) were male. Table 2 shows the demographical, epidemiological and tick transmission and infection predisposing factors. Most of the patients (82.9%) engaged in outdoor activities, mostly as a hobby, 39.0% had non-professional animal contact and 16.3% were farmers or cattle breeders. Only 26 patients recalled a recent tick bite and 20 of them recalled a tick removal. There were no patients with asplenia and only four had a known immunosuppressive condition. No differences between the Bb and BbBdv groups regarding demographical, epidemiological and predisposing factors were observed.

A complete list of patient clinical features and complications is presented in Table S1. Information on symptoms was available for 116 of the 120 patients, of whom 102 (87.9%) reported one or more symptoms associated with LB. The date of the infection was unknown in the few non-symptomatic patients (14 patients), although half of them had IgM antibodies against *Bo. burgdorferi* s.l., suggesting a recent infection.

Overall, 50% had neurological symptoms, rising to 55.6% in patients from the BbBdv group, 9.5% had unilateral and 2.2% bilateral facial paralysis and 18.2% erythema migrans. Osteomuscular symptoms were present in 37.8%, mostly arthralgias (31.1%), and 22.2% had constitutional symptoms, mostly fever (11.1%). Headache and gait disorders associated with both LB and babesiosis were observed in 13.3% and 11.1% of the patients of the BbBdv group, respectively. Other neurological symptoms associated with babesiosis such as confusion/delirium, impaired consciousness, ataxia and vision impairment [29] were rarely reported. In general, there were no differences between the two groups with regard to these clinical manifestations.

On the other hand, 13/116 (11.2%) had cardiorespiratory symptoms, with dyspnea as the most frequent (4.3%), see Table 3. Patients from the BbBdv group more frequently had cardiorespiratory symptoms compared to those of the Bb group (9/45 [20.0%] vs. 4/71 [5.6%], *p* = 0.02) especially dyspnea (4/45 [8.9%] vs. 1/71 [1.4%], *p* = 0.07).

Table 4 depicts the laboratory, radiological and ECG studies of the patients. Anaemia, or increased levels of lactate dehydrogenase (LDH) or bilirubin, parameters associated with haemolytic anaemia, were uncommon in both groups. Only five patients had Hgb levels < 11 g/dL, three of them from the BbBdv group, and one with anaemia-enhancing myelodysplastic syndrome. Normal median levels of C-reactive protein (CRP) or erythrocyte sedimentation rate (ESR) were also observed in both groups. Overall, 6 out of 69 (8.7%) patients had ECG abnormalities. Specifically, atrioventricular (AV) block was detected in 5 out of 32 (15.6%) patients in the BbBdv group, compared to one patient (1/37, 2.7%) in the Bb group (*p* = 0.09), in the absence of known prior heart disease. One of the patients in the BdBbv group had a second-degree AV block and the rest of the blocks were first-degree in both groups. None of the patients with cardiorespiratory symptoms had a previously known cardiopathy that might explain these symptoms. No differences regarding the manifestation of LB, its complications or the presence of an alternative diagnosis between the two groups were observed either. No other imaging or laboratory differences were observed between the two groups.

Regarding therapy, the most commonly administered antibiotic for *Bo. burgdorferi* s.l. was ceftriaxone (1.5–2.0 g/day), followed by doxycycline (200 mg/day) and amoxicillin (1500 mg/day), all for 2 weeks. None of these antibiotics have shown antibabesial activity. There were no significant differences between the two groups in any of the antimicrobial regimens administered.

On further assessment, the epidemiological, clinical and analytical features of patients from Bb and BbBdv groups, a forward stepwise logistic regression analysis model, including all variables with a *p* value ≤ 0.1 in the univariate analyses, revealed that the only significantly independent predictive factor for the BbBdv group was the presence of cardiorespiratory symptoms (OR 4.184, 95% CI 1.205–14.493, *p* = 0.024). Considering exclusively those patients who did not have these symptoms, the results were very similar to the overall population, both in the univariate and multivariate analyses. The only variable independently associated with the BbBdv group was again the existence of cardiorespiratory symptoms (OR 4.00, 95% CI 1.143–14.085, *p* = 0.03).

## 4. Discussion

In European regions such as Asturias (northwestern Spain), roe deer and cattle serve as hosts for the tick, *I. ricinus*, which transmits *Bo. burgdorferi* and *Ba. divergens*/*venatorum*, and these hosts are also reservoirs for *Ba. venatorum* and *Ba. divergens*, respectively. Furthermore, reported cases of both LB and human babesiosis suggest that these three pathogens are actively circulating in the area, posing a potential risk of infection to the local population [27,30,31,32,33].

However, limited information is available on individuals exposed to *Bo. burgdorferi* s.l. and *Ba. divergens/venatorum* pathogens. Indeed, efforts to differentiate *Ba. divergens* and *Ba. venatorum* infections using similar IFA systems and patient serum titration for serological diagnosis have demonstrated antigenic cross-reactivity [3,15,16]. This suggests that individuals with detectable antibodies may have been exposed to either or both *Babesia* species, especially in areas where both species have been detected in ticks and animals [9,10].

The occurrence of consecutive and simultaneous LB and human babesiosis in Europe remains poorly documented and largely unknown [19,20]. A recent meta-analysis found eight reports of *Bo. burgdorferi* s.l-*Babesia* spp. coinfection in the literature, all of them due to *Ba. microti* [34]. In the present study, after performing ECG in 57.5% of our patients, we observed that patients in the BbBdv group (5/32) had more cardiorespiratory symptoms, mainly dyspnea and AV block and dyspnea, than those in the Bb group (1/37). The cardiorespiratory symptoms were unrelated to anaemia and none of the patients with cardiorespiratory symptoms had previously reported heart disease able to explain these symptoms or ECG abnormalities. Cardiorespiratory symptoms were the only significant features that differed between the two groups.

This damage was manifested by ECG AV block in five patients, one of whom had second-degree AV block. A possible explanation for the cardiac dysfunction in the BbBdv group could be myocardial damage caused by both microorganisms, suggesting that both *Bo. burgdorferi* s.l. and *Ba. divergens/venatorum* could affect AV conduction and cause AV block. *Borrelia burgdorferi* AV block is a well-known complication of early disseminated LB [25]. Infections with *Babesia* species other than *Ba. divergens* have also been reported to cause cardiac abnormalities. For instance, *Babesia bigemina* infection induces changes in cardiac function biomarkers and D-dimer in cattle [35]. *Babesia canis* infection also induces cardiac disorders in dogs with a 32% rate of AV block [36]. Two reports showed myocardial damage and serious arrhythmia associated with severe babesiosis caused by *Ba. microti* in two American patients [37,38]. A recent report showed that 19.6% of hospitalized American patients with acute babesiosis due to *Ba. microti* developed cardiac complications, mostly atrial fibrillation, heart failure, QT interval prolongation and cardiac ischaemia [39]. No AV block of any degree was observed in that study, though. All the patients had high parasitaemias and had received antimicrobials (macrolides, quinine), which might have enhanced their heart arrhythmia. However, cardiac complications were not worse in those with confirmed *Bo. burgdorferi* s.l.-*Ba. microti* coinfection [39]. It is possible that *Ba. divergens/venatorum* subclinical or chronic infections could induce myocardial damage similar to that induced by *Ba. microti*. 

Limitations of this study include the retrospective study design, the fact that ECG was not performed in all patients, and the lack of other cardiac function biomarkers or echocardiogram tests that could have provided more data on cardiac complications. However, the full spectrum of cardiac complications associated with human babesiosis is unclear, including the aetiology and risk factors of cardiac pathology [39].

Another weakness of the study is the uncertainty of the timing of the infections. In this regard, we found that more than half of the patients in both groups had positive IgM serology for *Bo. burgdorferi* s.l., suggesting that they could have had a recent infection. In addition, there could be patients in both groups with a recent infection, but negative IgM serology if the blood extraction occurred before the time required to produce detectable antibodies. On the other hand, serology is not the diagnostic method of choice for babesiosis, because it does not distinguish between current and past infections [17].

Despite these limitations, the fact that a significant number of patients in this study were classified as infected with *Bo. burgdorferi* s.l. and seropositive for *Ba. divergens/venatorum*, including some with cardiorespiratory symptoms possibly due to both pathogens, is a notable finding. *Ba. divergens* and *Ba. venatorum* seroprevalence has been reported from asymptomatic patients [5,11,12,13,14] and may indicate long-term subclinical infections in which the pathogen persists without being fully cleared by the immune system, or a chronic infection characterized by intermittent periods of activity without overt clinical manifestations, as occurs with *Ba. microti* [5,11,12,13,14]. Further investigation is needed to better understand the clinical relevance of the presence of antibodies against species such as *Ba. divergens* and *Ba. venatorum* in the European population.

## 5. Conclusions

We conclude that patients who were infected with *Bo. burgdorferi* s.l. and with antibodies against *Ba. divergens/venatorum* from the BbBdv group developed cardiorespiratory symptoms significantly more commonly than patients infected with *Bo. burgdorferi* s.l. alone (Bb group). However, other symptoms, epidemiological factors and diagnostic tests did not differ between the two groups. While these patients were diagnosed and treated for LB, the diagnosis of babesiosis was not considered. Given that both infections share vectors and other epidemiological elements, babesiosis should be considered by physicians in patients with LB in co-endemic areas. It would also be desirable to develop reliable tests to establish the timing of the infections in patients infected with the three pathogens, in order to improve diagnosis and medical care, particularly for splenectomized and immunocompromised patients who are more prone to severe disease. In addition, more clinical investigation is needed to elucidate the unknown aspects of these combined infections in fully symptomatic patients, but also in those considered to be asymptomatic or paucisymptomatic.

## Figures and Tables

**Table 1:** Microbiological data from patients potentially exposed to *Ba. divergens/venatorum* infection in addition to *Bo. burgdorferi* s.l.

		Bb Group ^a^ (*n* = 73)	BbBdv Group ^b^ (*n* = 47)	*p* Value
*Borrelia burgdorferi* IgM serology	Positive	43 (58.9%)	27 (57.4%)	0.9
	Negative	30 (41.1%)	20 (42.6%)	
*Borrelia burgdorferi* IgG serology	Positive	73 (100%)	47 (100%)	-
	Negative	0 (0%)	0 (0%)	
*Borrelia burgdorferi* immunoblot	Positive	62 (88.6%)	39 (95.1%)	0.5
	Negative	1 (1.4%)	0 (0%)	
	Doubtful	7 (10.0%)	2 (4.9%)	
*Babesia divergens* indirect immunofluorescence assay	Positive	0 (0%)	47 (100%)	<0.0001
	Negative	73 (100%)	0 (0%)	
*Babesia divergens* protein extracts ^c^	Positive	0 (0%)	40 (85.1%)	<0.0001
	Negative	73 (100%)	7 (14.9%)	
GST-rBd37recombinant protein ^c^	Positive	0 (0%)	15 (31.9%%)	<0.0001
	Negative	73 (100%)	32 (68%)	

^a^ Bd group: patients infected with *Bo. burgdorferi* s.l.; ^b^ BbBdv group: patients infected with *Bo. burgdorferi* s.l. and with antibodies against *Ba. divergens/venatorum*; ^c^ *Ba. divergens* protein extracts and the purified Glutathione S-Transferase-tagged Bd37 recombinant protein (GST-rBd37) were used as target substrates for Western blot assays.

**Table 2:** Demography, epidemiology, and predisposing factors for patients potentially exposed to *Ba. divergens/venatorum* infection in addition to *Bo. burgdorferi* s.l.

		Bb Group ^a^ (*n* = 73)	BbBdv Group ^b^ (*n* = 47)	*p* Value
**Demography & epidemiology**				
Gender	Male	47 (64.4%)	34 (72.3%)	0.4
	Female	26 (35.6%)	13 (27.7%)	
Age years (*n* = 119)		58.0 (42.8–73.6)	56.4 (45.5–71.8)	0.8
Occupation	Farmer	4 (8.5%)	4 (12.1%)	0.2
	Breeder	3 (6.4%)	2 (6.1%)	
	Open air activity	0 (0%)	3 (9.1%)	
	Other	40 (85.1%)	24 (72.7)	
Tick bite identified	Yes	17 (24.6%)	9 (20.9%)	0.7
	No	52 (75.4%)	34 (79.1%)	
Tick removal	Yes	12 (17.4%)	8 (18.6%)	0.9
	No	57 (82.6%)	35 (81.4%)	
**Predisposing factors**	Yes	64 (90.4%)	42 (93.3%)	0.7
	No	7 (9.6%)	3 (6.7%)	
Transfusion	Yes	0 (0%)	0 (0%)	-
	No	69 (100%)	45 (100%)	
Asplenia	Yes	0 (0%)	0 (0%)	-
	No	69 (100%)	45 (100%)	
Immunosuppression	Yes	2 (2.9%)	2 (4.4%)	0.6
	No	68 (97.1%)	43 (95.6%)	
Age > 50 years	Yes	43 (59.7%)	33 (70.2%)	0.2
	No	29 (40.3%)	14 (29.8%)	
Outdoor hobbies	Yes	45 (72.6%)	34 (82.9%)	0.2
	No	17 (27.4%)	7 (17.1%)	
Non-professional animal contact	Yes	18 (29.0%)	16 (39.0%)	0.3
	No	44 (71.0%)	25 (61.0%)	

Values are expressed as median (IQ range) or %. ^a^ Bd group: patients infected with *Bo. burgdorferi* s.l.; ^b^ BbBdv group: patients infected with *Bo. burgdorferi* s.l. and with antibodies against *Ba. divergens/venatorum*.

**Table 3:** Cardiorespiratory symptoms potentially exposed to *Ba. divergens/venatorum* infection in addition to *Bo. burgdorferi* s.l.

		Bb Group ^a^ *(n* = 71)	BbBdv Group ^b^ (*n* = 45) ^c^	*p* Value
**Cardiorespiratory symptoms ^c^**	Yes	4 (5.6%)	9 (20.0%)	0.02
	No	67 (94.4%)	36 (80.0%)	
Syncope	Yes	1 (1.4%)	2 (4.4%)	0.6
	No	70 (98.6%)	43 (95.6%)	
Chest pain	Yes	2 (2.8%)	3 (6.7%)	0.4
	No	69 (97.2%)	42 (93.3%)	
Dyspnea	Yes	1 (1.4%)	4 (8.9%)	0.07
	No	70 (98.6%)	41 (91.1%)	
Palpitations	Yes	0 (0%)	0 (0%)	-
	No	71 (100%)	45 (100%)	

Values are expressed as median (IQ range) or %. ^a^ Bd group: patients infected with *Bo. burgdorferi* s.l.; ^b^ BbBdv group: patients infected with *Bo. burgdorferi* s.l. and with antibodies against *Ba. divergens/venatorum*. ^c^ Cardiorespiratory symptoms’ information was available in only 45 patients of the BbBdv group.

**Table 4:** Imaging, electrocardiographic (ECG) and laboratory studies ^a^.

		Bb Group ^b^ (*n* = 71)	BbBdv Group ^c^ (*n* = 47)	*p* Value
Chest X-ray	Normal	38 (97.4%)	26 (92.9%)	0.6
	Abnormal	1 (2.6%)	2 (7.1%)	
ECG	Normal	36 (97.3%)	27 (84.4%)	0.09
	AV block ^d^	1 (2.7%)	5 (15.6%)	
**Laboratory blood determinations **				
Total leukocyte counts	cells/μL (*n* = 109)	7500.0 (6165.0–9065.0)	7240.0 (5612.5–9520.0)	0.6
Absolute neutrophil counts	cells/μL (*n* = 109)	4180.0 (3170.0–5765.0)	4520.0 (2885.0–6552.5)	0.8
Absolute lymphocyte counts	cells/μL (*n* = 107)	1940.0 (1460.0–2590.0)	1740.0 (1470.0–2307.5)	0.3
Absolute eosinophil counts	cells/μL (*n* = 106)	130.0 (77.5–232.5)	135.0 (72.5–245.0)	0.9
Platelets	per μL (*n* = 109)	232,000 (183,000–294,000)	236,000 (200,250–285,250)	1
Haemoglobin	gr/dL (*n* = 109)	14.10 (13.40–15.20)	14.40 (13.38–15.23)	0.8
C-reactive protein	mg/L (*n* = 89)	0.350 (0.100–0.925)	0.300 (0.100–1.400)	0.7
Erythrocyte sedimentation rate	mm/h (*n* = 66)	12.0 (4.8–22.0)	11.0 (5.0–29.0)	0.6
Aspartate aminotransferase	U/L (*n* = 37)	24.0 (19.0–36.3)	21.0 (20.0–28.0)	0.9
Alanine aminotransferase	U/L (*n* = 93)	19.0 (15.0–31.0)	23.0 (17.0–28.0)	0.2
Lactate dehydrogenase	U/L (*n* = 39)	208.0 (182.0–237.0)	207.0 (168.8–254.5)	0.9
Total bilirubin	mg/dL (*n* = 73)	0.90 (0.90–0.90)	0.90 (0.90–0.90)	0.3
Alkaline phosphatase	U/L (*n* = 89)	73.0 (59.0–87.0)	62.0 (52.5–83.3)	0.2
Creatinine	mg/dL (*n* = 106)	0.85 (0.74–1.02)	0.80 (0.74–0.93)	0.4

Values are expressed as median (IQ range) or %. ^a^ Clinical, laboratory and complementary data were available for 116 /120 patients enrolled in the study; ^b^ Bd group: patients infected with *Bo. burgdorferi* s.l.; ^c^ BbBdv group: patients infected with *Bo. burgdorferi* s.l. and with antibodies against *Ba. divergens/venatorum*; ^d^ a patient from the BbBdv group had second-degree AV block, whereas the remaining patients had first-degree block.

## Data Availability

The original contributions presented in this study are included in the article/Supplementary Material. Further inquiries can be directed to the corresponding author.

## Supplementary Materials

The following supporting information can be downloaded at: https://www.mdpi.com/article/10.3390/microorganisms13061383/s1, Table S1. Clinical features and complications.
