# Supplementary material for: Epidemiological, Clinical and Analytical Features in Lyme Borreliosis Patients Seropositive for Babesia divergens/venatorum
Source: Microorganisms. 2025 Jun 13;13(6):1383. doi: 10.3390/microorganisms13061383 (PMC12195485; doi:10.3390/microorganisms13061383)
Supplement: Supplementary file 1 [file microorganisms-13-01383-s001.zip › microorganisms-3647513-supplementary.pdf]

Table S1. Clinical features and complications [a].

		**Bb group[b] (*n*=71)**	**BbBdv group[c] (*n*=47)**	**P value**
**SYMPTOMS**				
Duration of symptoms	days (n=81)	15.0 (4.0-67.5)	15.00 (7.00-120.0)	0.3
One or more symptoms	Yes	61 (85.9%)	41 (91.1%)	0.9
	No	10 (14.1%)	4 (8.9%)	
**Constitutional symptoms**	Yes	15 (21.1%)	10 (22.2%)	0.9
	No	56 (78.9%)	35 (77.8%)	
Fever	Yes	9 (12.7)	5 (11.1%)	0.8
	No	62 (87.3%)	40 (88.9%)	
Asthenia	Yes	8 (11.3%)	6 (13.3%)	0.7
	No	63 (88.7%)	39 (86.7%)	
Anorexia	Yes	2 (2.8%)	0 (0%)	0.5
	No	69 (97.2%)	45 (100%)	
Weight loss	Yes	0 (0%)	0 (0%)	-
	No	71 (100%)	45 (100%)	
**Osteomuscular symptoms**	Yes	22 (31.0%)	17 (37.8%)	0.5
	No	49 (69.0%)	28 (62.2%)	
Arthralgias	Yes	19 (26.8%)	14 (31.1%)	0.6
	No	52 (73.2%)	31 (68.9%)	
Arthritis	Yes	12 (16.9%)	5 (11.1%)	0.4
	No	59 (83.1%)	40 (88.9%)	
Myalgias	Yes	7 (9.9%)	9 (20.0%)	0.12
	No	64 (90.1%)	36 (80.0%)	
**Digestive symptoms**	Yes	3 (4.2%)	0 (0%)	0.3
	No	68 (95.8%)	45 (100%)	
Abdominal pain	Yes	2 (2.8%)	0 (0%)	0.5
	No	69 (97.2%)	45 (100%)	
Nausea	Yes	0 (0%)	0 (0%)	-
	No	71 (100%)	45 (100%)	
Vomiting	Yes	1 (1.4%)	0 (0%)	1
	No	70 (98.6%)	45 (100%)	
Diarrhoea	Yes	0 (0%)	0 (0%)	-
	No	71 (100%)	45 (100%)	
**Cardiorespiratory symptoms***	Yes	4 (5.6%)	9 (20.0%)	0.02
	No	67 (94.4%)	36 (80.0%)	
Syncope	Yes	1 (1.4%)	2 (4.4%)	0.6
	No	70 (98.6%)	43 (95.6%)	
Chest pain	Yes	2 (2.8%)	3 (6.7%)	0.4
	No	69 (97.2%)	42 (93.3%)	
Dyspnoea	Yes	1 (1.4%)	4 (8.9%)	0.07
	No	70 (98.6%)	41 (91.1%)	
Palpitations	Yes	0 (0%)	0 (0%)	-

	No	71 (100%)	45 (100%)	
**Neurological symptoms**	Yes	33 (46.5%)	25 (55.6%)	0.3
	No	38 (53.5%)	20 (44.4%)	
Loss of strength	Yes	11 (15.5%)	9 (20.0%)	0.5
	No	60 (84.5%)	36 (80.0%)	
Gait disturbance	Yes	8 (11.3%)	5 (11.1%)	1
	No	63 (88.7%)	40 (88.9%)	
Cranial nerve involvement	Yes	7 (9.9%)	3 (6.7%)	0.7
	No	64 (90.1%)	42 (93.3%)	
Paresthesia	Yes	8 (11.3%)	7 (15.6%)	0.5
	No	63 (88.7%)	38 (84.4%)	
Dizziness	Yes	3 (4.3%)	2 (4.4%)	1
	No	67 (95.7%)	43 (95.6%)	
Headache	Yes	13 (18.3%)	6 (13.3%)	0.5
	No	58 (81.7%)	39 (86.7%)	
Hyperesthesia	Yes	0 (0%)	1 (2.2%)	0.4
	No	71 (100%)	44 (97.8%)	
Other symptoms	Yes	9 (12.7%)	4 (8.9%)	0.5
	No	62 (87.3%)	41 (91.1%)	
**Ophthalmological symptoms**	Yes	3 (4.2%)	1 (2.2%)	1
	No	68 (95.8%)	44 (97.8%)	
Photophobia	Yes	2 (2.8%)	1 (2.2%)	1
	No	69 (97.2%)	44 (97.8%)	
**Cutaneous symptoms**	Yes	18 (25.4%)	13 (28.9%)	0.7
	No	53 (74.6%)	32 (71.1%)	
Erythema migrans	Yes	14 (19.7%)	8 (18.2%)	0.8
	No	57 (80.3%)	36 (81.8%)	
**Other symptoms**	Yes	9 (12.7%)	1 (2.2%)	0.09
	No	62 (87.3%)	44 (97.8%)	
**PHYSICAL EXAM**				
**General**	Yes	2 (2.8%)	2 (4.4%)	0.6
	No	69 (97.2%)	43 (95.6%)	
Jaundice	Yes	0 (0%)	0 (0%)	-
	No	71 (100%)	45 (100%)	
Pharyngeal erythema	Yes	0 (0%)	1 (2.2%)	0.4
	No	71 (100%)	44 (97.8%)	
Lymphadenopathy	Yes	2 (2.8%)	2 (4.4%)	0.6
	No	69 (97.2%)	43 (95.6%)	
Hepatomegaly	Yes	0 (0.0%)	1 (2.2%)	0.4
	No	71 (100%)	44 (97.8%)	
Splenomegaly	Yes	0 (0.0%)	1 (2.2%)	0.4
	No	71 (100%)	44 (97.8%)	
**Neurological**	Yes	17 (23.9%)	11 (24.4%)	0.9
	No	54 (76.1%)	34 (75.6%)	
Meningeal signs	Yes	2 (2.8%)	0 (0%)	0.5

	No	69 (97.2%)	45 (100%)	
Nystagmus	Yes	2 (2.8%)	0 (0%)	0.5
	No	69 (97.2%)	45 (100%)	
Unilateral facial paralysis	Yes	8 (11.3%)	3 (6.7%)	0.5
	No	63 (88.7%)	42 (93.3%)	
Bilateral facial paralysis	Yes	2 (2.8%)	1 (2.2%)	1
	No	69 (97.2%)	44 (97.8%)	
Other cranial nerve involvement	Yes	2 (2.8%)	0 (0%)	0.5
	No	69 (97.2%)	45 (100%)	
Romberg sign	Yes	1 (1.4%)	0 (0%)	1
	No	70 (98.6%)	45 (100%)	
Babinski sign	Yes	2 (2.8%)	1 (2.2%)	1
	No	69 (97.2%)	44 (97.8%)	
Muscle weakness	Yes	12 (16.9%)	7 (15.6%)	0.8
	No	59 (83.1%)	38 (84.4%)	
**Ophthalmological**	Yes	1 (1.4%)	1 (2.2%)	1
	No	70 (98.6%)	44 (97.8%)	
Retinal infarction	Yes	0 (0%)	0 (0%)	-
	No	71 (100%)	45 (100%)	
Retinal haemorrhage	Yes	0 (0%)	0 (0%)	-
	No	71 (100%)	45 (100%)	
Conjunctival hyperaemia	Yes	1 (1.4%)	1 (2.2%)	1
	No	70 (98.6%)	44 (97.8%)	
**LYME BORRELIOSIS STAGE**				
Early localised Lyme borreliosis	Yes	22 (31.0%)	14 (31.1%)	0.9
	No	49 (69.0%)	31 (68.9%)	
Early disseminated Lyme borreliosis	Yes	12 (16.9%)	7 (15.6%)	0.8
	No	59 (83.1%)	38 (84.4%)	
Late Lyme borreliosis	Yes	3 (4.2%)	3 (6.7%)	0.7
	No	68 (95.8.%)	42 (93.3%)	
Neurological Lyme borreliosis [d]	Yes	12 (16.9%)	7 (15.6%)	0.8
	No	59 (83.1%)	38 (84.4%)	
Past infection by *B.burgdorferi*	Yes	26 (36.6%)	17 (37.8%)	0.9
	No	45 (63.4%)	28 (62.2%)	
Unknown stage		7 (9.9%)	6 (12.8%)	
**COMPLICATIONS**	Yes	0 (0.0%)	1 (2.2%)	0.4
	No	71 (100%)	44 (97.8%)	
Respiratory distress	Yes	0 (0%)	0 (0%)	-
	No	71 (100%)	45 (100%)	
Heart failure	Yes	0 (0.0%)	1 (2.2%)	0.4
	No	71 (100%)	44 (97.8%)	
Disseminated intravascular coagulation	Yes	0 (0%)	0 (0%)	-
	No	71 (100%)	45 (100%)	
Splenic infarct	Yes	0 (0%)	0 (0%)	-
	No	71 (100%)	45 (100%)	

Splenic rupture	Yes	0 (0%)	0 (0%)	-
	No	71 (100%)	45 (100%)	
Other complications	Yes	0 (0%)	0 (0%)	-
	No	71 (100%)	45 (100%)	

Values are expressed as median (IQ range) or %. [a] Clinical, laboratory and complementary data were available for 116 /120 patients enrolled in the study; [b] Bd group: patients infected with *B. burgdorferi* s.l.; [c] BbBdv group: patients infected with *Bo. burgdorferi* s.l. and with antibodies against *Ba. divergens/venatorum*; [d] Patients with neurological Lyme borreliosis were included among those with early disseminated Lyme borreliosis; *Cardiorespiratory symptoms are also also presented as Table 3.
